# Analysis of the Relationship Between the Degree of Dysbiosis in Gut Microbiota and Prognosis at Different Stages of Primary Hepatocellular Carcinoma

**DOI:** 10.3389/fmicb.2019.01458

**Published:** 2019-06-25

**Authors:** Jiajia Ni, Rong Huang, Huifang Zhou, Xiaoping Xu, Yang Li, Peihua Cao, Kebo Zhong, Mei Ge, Xiaoxia Chen, Baohua Hou, Min Yu, Baogang Peng, Qiao Li, Peng Zhang, Yi Gao

**Affiliations:** ^1^Department of Hepatobiliary Surgery II, Guangdong Provincial Research Center of Artificial Organ and Tissue Engineering, Zhujiang Hospital of Southern Medical University, Guangzhou, China; ^2^State Key Laboratory of Organ Failure Research, Southern Medical University, Guangzhou, China; ^3^Department of Neonatal Surgery, Guangdong Women and Children Hospital, Guangzhou, China; ^4^Department of Clinical Laboratory, First People’s Hospital of Kashi, Kashgar, China; ^5^Department of General Surgery, Guangdong General Hospital, Guangdong Academy of Medical Sciences, Guangzhou, China; ^6^Department of Hepatic Surgery, The First Affiliated Hospital, Sun Yat-sen University, Guangzhou, China; ^7^Department of Organ Transplantation, The Second Affiliated Hospital of Guangzhou Medical University, Guangzhou, China

**Keywords:** chronic liver diseases, dysbiosis degree, gut microbiota, primary hepatocellular carcinoma, prognosis

## Abstract

Gut microbiota dysbiosis is closely associated with primary hepatocellular carcinoma (HCC). Recent studies have evaluated the early diagnosis of primary HCC through analysis of gut microbiota dysbiosis. However, the relationship between the degree of dysbiosis and the prognosis of primary HCC remains unclear. Because primary HCC is accompanied by dysbiosis and dysbiosis usually increases the circulatory concentrations of endotoxin and other harmful bacterial substances, which further increases liver damage, we hypothesized that level of dysbiosis associated with primary HCC increases with the stage of cancer progression. To test this hypothesis, we introduced a more integrated index referred to as the degree of dysbiosis (*D_dys_*); and we investigated *D_dys_* of the gut microbiota with the development of primary HCC through high-throughput sequencing of 16S rRNA gene amplicons. Our results showed that compared with healthy individuals, patients with primary HCC showed increased pro-inflammatory bacteria in their fecal microbiota. The *D_dys_* increased significantly in patients with primary HCC compared with that in healthy controls. Moreover, there was a tendency for the *D_dys_* to increase with the development of primary HCC, although no significant difference was detected between different stages of primary HCC. Our findings provide important insights into the use of gut microbiota analysis during the treatment of primary HCC.

## Introduction

Disruption of the gut microbiota (termed “dysbiosis”) is closely associated with the development of chronic liver diseases (CLDs) in humans and rodent models (Aron-Wisnewsky et al., 2013; Grice and Segre, 2013; Kamada et al., 2013; Schnabl, 2013; Bajaj et al., 2014a; Grąt et al., 2016; Houghton et al., 2016; Shen et al., 2017). Several studies have reported that dysbiosis is associated with CLDs of different etiologies (Quigley et al., 2013; Schnabl, 2013; Xie et al., 2016). For example, patients with chronic hepatitis or decompensated cirrhosis secondary to hepatitis B infection showed reduced numbers of probiotic Bifidobacteria and lactic acid-producing bacteria in the feces, whereas *Enterococcus faecalis* and Enterobacteriaceae numbers were higher than those in asymptomatic carriers and healthy controls (Lu et al., 2011). In addition, using deep high-throughput sequencing of the 16S rRNA gene of bacteria, fecal microbial communities from patients with alcoholic or hepatitis B-related cirrhosis could be clearly distinguished from healthy controls. Increases in Streptococcaceae, Veillonellaceae, and Enterobacteriaceae, accompanied by a decrease in Lachnospiraceae, characterize the gut microbiota in cirrhosis. The relative abundances of the Lachnospiraceae and Streptococcaceae families were negatively and positively related with the Child-Pugh score in patients with cirrhosis, respectively (Chen et al., 2011).

Hepatocellular carcinoma (HCC) is a type of advanced CLD and a long-term consequence of chronic liver injury, inflammation, and fibrosis (Darnaud et al., 2013). HCC is the third leading cause of cancer-related death worldwide (El-Serag and Kanwal, 2014; Yu and Schwabe, 2017). Approximately 29,200 new HCC cases in men and 11,510 new HCC cases in women were reported in the United States of America (USA) in 2017 (Siegel et al., 2017). The incidence in China is worse, with new HCC cases reported in more than 343,000 men and 122,000 women in 2015 (Chen et al., 2016). Gut microbiota dysbiosis is closely associated with HCC, and recently, Ren et al. (2018) reported that 13 genera, including *Gemmiger* and *Parabacteroides*, were enriched in early HCC following progression from cirrhosis to HCC.

Dysbiosis accompanies the occurrence of HCC and usually increases the circulatory concentrations of endotoxin and other harmful bacterial substances, which further increases liver damage (Yu et al., 2010; Darnaud et al., 2013; Yu and Schwabe, 2017; Ma et al., 2018). Therefore, we hypothesized that level of dysbiosis associated with primary HCC increases with the stage of cancer progression, suggesting clinical importance in the prognosis of patients with HCC.

Although dysbiosis is commonly reported in fecal microbiota from patients with HCC and several methods are available to evaluate gut bacterial taxa and quantify the degree of dysbiosis in patients with other CLDs (Lu et al., 2011; Wong et al., 2013; Bajaj et al., 2014b; Grąt et al., 2015), still no viable integrated approach to measure the degree of dysbiosis in patients with HCC. Therefore, it is difficult to assess whether the dysbiosis becomes more serious with the progression of HCC. This study introduced a novel integrated index that could be utilized to determine the degree of dysbiosis in HCC patients. This new index is called the degree of dysbiosis (*D_dys_*) and used to identify disparities in the gut microbiota during the development of HCC, via high-throughput sequencing of 16S rRNA gene amplicons. Our findings provide important insights into the use of gut microbiota analysis during the treatment of HCC.

## Materials and Methods

### Inclusion and Exclusion Criteria

The study protocol was approved by the Medical Ethics Committee of Zhujiang Hospital, Southern Medical University (approval number: 2017-GDEK-002) and was performed in accordance with clinical ethics guidelines and the Declaration of Helsinki and Rules of Good Clinical Practice. Patients with primary HCC were age-matched to healthy controls and recruited from five hospitals in Guangzhou, a large modern city in southern China. All patients and healthy controls provided informed consent for their participation in the study. No specific medical intervention was conducted specifically for this study.

Patients with primary HCC were defined as having histological evidence, radiological evidence, or clinical diagnosis of primary HCC (Ye, 2009). Primary HCC samples were staged according to the National Health and Family Planning Commission of the People’s Republic of China (Zhou et al., 2018). We excluded patients with an unclear diagnosis of primary HCC, those with inflammatory bowel disease, those with a current infection, those on gut-absorbable antibiotic therapy, those with type 2 diabetes or hypertension, and those with incomplete clinically diagnostic information. If the above criteria were met, participants were enrolled in the study and admitted to hospital following diagnosis. No other therapy was provided to patients in the 2 months before fecal sampling. Analysis of the fecal microbiota profiles was based on prospectively collected stool samples from the pretherapy period and immediately stored at −80°C. The patients were recruited based on their admission time in the hospital during the study period August 1, 2017 to November 30, 2017.

We only included age-matched healthy controls without clinically diagnosable disease and those who had not taken antibiotics or probiotics in the last 2 months. The healthy controls were recruited as volunteers for the out-patient physical examinations occurring during the study period. Analysis of the fecal microbiota profiles was conducted following the initial clinical examination and participants lacking clinically diagnosable disease were accepted for the study as healthy volunteers, and their stool samples were subjected to further analysis.

Two reduplicate fecal samples were collected using fecal collectors synchronously from each participant; one sample was used to analyze microbial composition and the other reserved for further testing.

### Fecal DNA Extraction and High-Throughput Sequencing

Fresh fecal pellets (0.3 g) of each participant were used for microbial DNA extraction. Fecal microbial DNA was extracted using a PowerSoil DNA isolation kit (Mobio, United States). DNA concentration and quality were checked using a NanoDrop spectrophotometer (Thermo Fisher Scientific, United States).

The V4–V5 hypervariable region of the prokaryotic 16S rRNA gene was amplified using the universal primer pair 515F (5′-GTGYCAGCMGCCGCGGTA-3′) and 909R (5′-CCCCGYCAATTCMTTTRAGT-3′), with a 12-nt sample-specific barcode sequence included at the 5′-end of the 515F sequence to distinguish samples (Ni et al., 2017; Huang et al., 2018; Xiang et al., 2018). Polymerase chain reaction (PCR) was performed, and amplicons were sequenced using a MiSeq system at Guangdong Meilikang Bio-Science, Ltd. (China), as described previously (Huang et al., 2018; Xiang et al., 2018).

The raw sequences were merged using FLASH-1.2.8 software (Magoc and Salzberg, 2011) and processed using the QIIME Pipeline 1.9.0 with default parameters (Caporaso et al., 2010). Chimeric sequences were identified and removed using the Uchime algorithm before further analysis (Edgar et al., 2011). The high-quality sequences were clustered into OTUs at 97% identity using UPARSE (Edgar, 2013). Taxonomic assignments of each OTU were determined using the RDP classifier (Wang et al., 2007).

### Definition of the Degree of Dysbiosis

In order to quantify the degree of dysbiosis in patients with primary HCC, we compared the ratio of abundance of Firmicutes to Bacteroidetes (Wong et al., 2013), the ratio of abundance of autochthonous taxa to non-autochthonous taxa (Bajaj et al., 2014b), and the ratio of abundance of the genus *Bifidobacterium* to the family Enterobacteriaceae (Lu et al., 2011) at different stages of primary HCC and healthy controls, as these ratios were reported associated with other liver diseases. In addition, we introduced a more integrated index for measuring the dysbiosis. This index was calculated based on the relative abundance of seven types of inherently probiotic bacterial genera with decreased abundance in the fecal microbiota of patients with CLDs (*Anaerostipes*, *Bifidobacterium*, *Coprococcus*, *Faecalibacterium*, *Lactobacillus*, *Oscillibacter*, and *Phascolarctobacterium*) and 13 potentially harmful bacterial genera that generally increased in the fecal microbiota of these patients (*Akkermansia*, *Bacteroides*, *Clostridium*, *Dorea*, *Escherichia*, *Fusobacterium*, *Haemophilus*, *Helicobacter*, *Klebsiella*, *Prevotella*, *Ruminococcus*, *Streptococcus*, and *Veillonella*) (Huang et al., 2004; Fox et al., 2010; Malaguarnera et al., 2010; Zhang et al., 2012; Ren et al., 2018; Zmora et al., 2018). The degree of dysbiosis was then calculated according to the follow formula:

$$\begin{array}{c} D_{\mathrm{dys}}=\Sigma (\log_{10}[100\times {\mathrm{RA}}_{\mathrm{harmful}}+1])\\ \;-\Sigma (\log_{10}[100\times {\mathrm{RA}}_{\mathrm{Probiotic}}+1]) \end{array}$$

where *D_dys_* was the degree of dysbiosis; *RA_harmful_* was the relative abundance of each harmful bacterial genus; and *RA_probiotic_* was the relative abundance of each probiotic bacterial genus.

### Statistical Analysis

The results for each parameter are presented as the mean ± standard error for each group. Non-parametric Adonis tests (Anderson, 2001) were applied to test the significance of differences among three or more groups using the R vegan package (Dixon, 2003). The indicator value method (McGeoch, 2007) was used to screen potentially significantly different genera among the groups. The values were calculated through the R indicspecies package. Screened genera were verified using the standard non-parametric Kruskal–Wallis test through R with the ggpubr package according to a previous report (Li et al., 2018). The Kruskal–Wallis test was also used to detect the statistical significance of alpha-diversity indices among patients with different stages of HCC and healthy controls. Cladogram layout was drawn using GraPhlAn software (Asnicar et al., 2015). Box plots were drawn to show the relative abundances of significantly different dominant phyla or genera among groups using R software with the ggpubr package. Statistically significant markers were added to the box plots using Adobe Illustrator CS5 software according to the Wilcoxon rank sum test results. Correlation analyses were conducted using R software. Results with *P*-values of less than or equal to 0.05 were considered statistically significant.

### Availability of Data

The merged DNA datasets were deposited in the NCBI Sequence Read Archive database (accession number SRP151835).

## Results

### Baseline Characteristics Examined for All Volunteers

In total, 110 fecal samples were prospectively collected from 110 participants and subjected to MiSeq sequencing, and after a strict exclusion process, 86 samples (23 cases of stage I of primary HCC; 13 cases of stage II of primary HCC; 30 cases of stage III of primary HCC; 2 cases of stage IV of primary HCC; and 18 healthy control individuals) were finally included for further analysis (Table 1); other samples did not meet the inclusion criteria. The samples from patients with stages III and IV HCC were grouped together because these stages were considered advanced HCC. No significant differences were detected between the different stages of primary HCC and the healthy controls with regard to age (Kruskal–Wallis test, χ^2^ = 7.62, *p* = 0.054), height (Kruskal–Wallis test, χ^2^ = 1.63, *p* = 0.652), weight (Kruskal–Wallis test, χ^2^ = 4.64, *p* = 0.200), body mass index (BMI, Kruskal–Wallis test, χ^2^ = 7.29, *p* = 0.063), and systolic pressure (SP, Kruskal–Wallis test, χ^2^ = 4.45, *p* = 0.217). However, fasting blood glucose (FBG, Kruskal–Wallis test, χ^2^ = 12.62, *p* = 0.006) and albumin (Kruskal–Wallis test, χ^2^ = 19.02, *p* < 0.001) were significantly lower in primary HCC cases with different stages than healthy controls, and alanine aminotransferase (ALT) was significantly higher (Kruskal–Wallis test, χ^2^ = 14.99, *p* = 0.002). In addition, total bilirubin (TB) was significantly higher in stage III of primary HCCs than the healthy controls (Table 1).

**Table 1:** Basic and physiological data of patients with primary HCC and healthy controls.

Sample ID	HCC-I	HCC-II	HCC-III	HCC-IV^∗^	Healthy

Specimen number	23	13	30	2	18
Age	52.96 ± 2.49	59.31 ± 2.06	52.47 ± 1.41		50.28 ± 2.28
Height (cm)	163.65 ± 1.42	166.38 ± 1.00	165.22 ± 1.25		165.17 ± 1.71
Weight (kg)	63.04 ± 2.23	60.08 ± 2.42	61.88 ± 1.53		65.33 ± 2.54
Body mass index	23.47 ± 0.67	21.73 ± 0.92	22.64 ± 0.45		23.90 ± 0.80
Systolic pressure	122.21 ± 3.94	124.46 ± 5.06	127.34 ± 2.58		134.72 ± 4.68
Fasting blood glucose (mmol/L)	5.08 ± 0.29^a^	4.79 ± 0.46^a^	4.74 ± 0.15^a^		5.83 ± 0.43^b^
Total bilirubin (μmol/L)	17.97 ± 2.46^a^	21.64 ± 3.04^ab^	42.94 ± 8.85^b^		12.46 ± 1.07^a^
Albumin (g/L)	37.34 ± 1.39^a^	36.46 ± 0.87^a^	35.21 ± 1.04^a^		41.53 ± 0.85^b^
Alanine aminotransferase (IU/L)	51.21 ± 6.84^a^	50.77 ± 6.76^a^	66.40 ± 11.86^a^		27.17 ± 5.19^b^

*Primary HCC samples were staged as previously described (Zhou et al., 2018). Results with P-values of less than or equal to 0.05 were considered statistically significant. Lowercases at the upper right corner of the mean ± standard error showed the statistical significance. ^∗^The samples from patients with stages III and IV HCC were mixed together for further analysis because these stages were considered advanced HCC.*

### Proteobacteria Were Increased in the Gut Microbiota of Patients With Primary HCC

**FIGURE 1:**
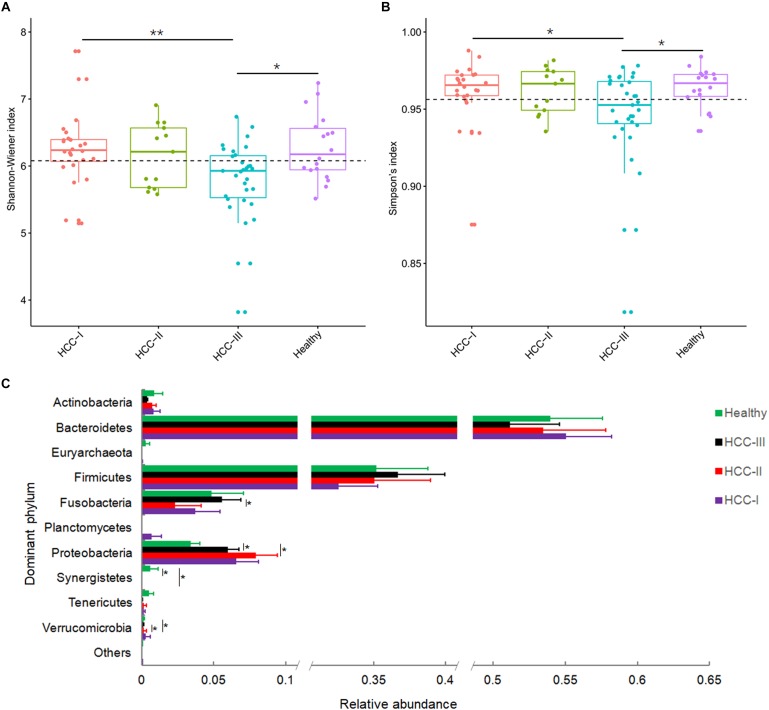
Shannon-Wiener index **(A)**, Simpson’s index **(B)**, and relative abundances of the dominant phyla of fecal microbiota from patients with primary hepatocellular carcinoma (HCC) and healthy controls **(C)**. Patients with primary HCC were staged as described previously (Zhou et al., 2018). ^∗∗^*p* < 0.01; ^∗^*p* < 0.05.

After low-quality and chimeric sequences were removed, 5,258,105 (61,140.76 ± 2,723.202) high-quality sequences were obtained. To eliminate the influence of sequencing depth, 20,293 sequences were randomly sampled for further analysis. In total, 7,655 operational taxonomic units (OTUs) from 604 genera were identified at 97% sequence similarity. Although the alpha-diversities of the microbiota from advanced primary HCC (stage III and IV primary HCC) were significantly reduced compared with that in healthy controls (Figure 1A,B), no significant differences were detected between early primary HCC and healthy controls (Figure 1A,B), consistent with a previous report (Ren et al., 2018), although they found that microbial diversity was markedly increased in early primary HCC versus liver cirrhosis. The OTUs belonged to 38 phyla, with the exception of tiny unclassified sequences (0–0.19%, 0.004 ± 0.002%). However, only 10 phyla, i.e., Actinobacteria, Bacteroidetes, Euryarchaeota, Firmicutes, Fusobacteria, Planctomycetes, Proteobacteria, Synergistetes, Tenericutes, and Verrucomicrobia, were the dominant phyla, with relative abundances of more than 1% in at least one sample (Figure 1C and Supplementary Figure S1). These phyla accounted for up to 99.92 ± 0.01% of the analyzed high-quality sequences. Although the relative abundances of *Firmicutes* were not significantly changed in patients with primary HCC, these *Proteobacteria* were significantly increased in patients with stages II and III primary HCC compared with that in healthy controls (Figure 1C). Because most pro-inflammatory bacteria come from *Proteobacteria* and many probiotic bacteria come from *Firmicutes* (Stecher et al., 2013; Gao et al., 2015), this result implied that pro-inflammatory bacteria accompanied the development of primary HCC. Simultaneously, many pro-inflammatory bacteria in *Proteobacteria*, such as those of Enterobacteriaceae, were indicators of dysbiosis. Therefore, dysbiosis may worsen with the progression of primary HCC. To determine which bacterial species led to the expansion of *Proteobacteria*, we analyzed the gut microbiota at the genus level.

### The Degree of Dysbiosis Increased in Patients With Primary HCC

**FIGURE 2:**
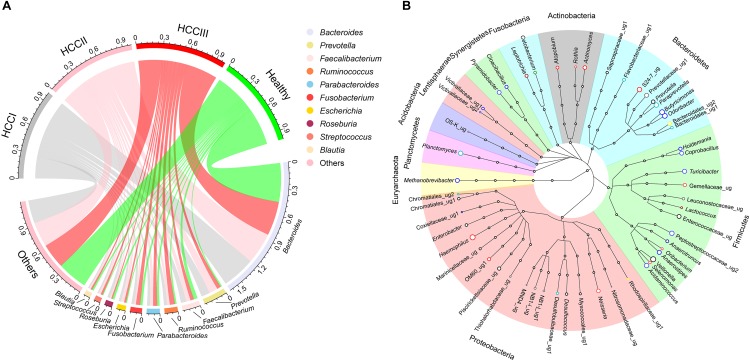
Circular layout **(A)** and Cladogram layout **(B)** depict the top 10 genera found in the fecal microbiota, as well as significantly different genera found in the gut microbiota among patients with different primary HCC stages and the healthy controls. Primary HCC samples were staged as previously described (Zhou et al., 2018). Circle colors in the layout **(B)** show changes in genera within the fecal microbiota of patients expressing different stages of primary HCC, compared with healthy controls. The genera that were enhanced include yellow (stage I), gray (stage II), light red (stage III), pink (stages I and II), red (stages I and III), dark red (stages II and III), and black (stages I, II and III). The genera that were reduced by color bright green (stage I), green (stage II), blue (stage III), pale green (stages I and II), light blue (stages I and III), dark blue (stage II and III), and dark green (stages I, II and III), respectively.

There were 604 genera identified from the 86 fecal samples. *Bacteroides* (39.91 ± 2.01%) was the most dominant genus, followed by *Prevotella* (6.19 ± 1.36%), *Faecalibacterium* (4.83 ± 0.50%), *Ruminococcus* (4.07 ± 0.52%), *Parabacteroides* (3.79 ± 0.43%), *Fusobacterium* (3.58 ± 0.70%), *Escherichia* (2.34 ± 0.43%), *Roseburia* (2.08 ± 0.26%), *Streptococcus* (1.84 ± 0.41%), and *Blautia* (1.76 ± 0.19%). The top 10 genera accounted for up to 71.75 ± 1.63% of the analyzed high-quality sequences (Figure 2A). In total, 54 genera were found to be significantly different among groups at the various primary HCC stages or the healthy controls based on the indicator value and the standard non-parametric Kruskal–Wallis test. Compared with healthy controls, *Actinomyces*, *Atopobium*, *Desulfococcus*, *Enterobacter*, *Paraprevotella*, *Planctomycetes*, *Prevotella*, *Veillonella* and many unidentified genera were enhanced in patients with stage I HCC. *Desulfococcus*, *Enterobacter*, *Lactococcus*, *Leptotrichia*, *Paraprevotella*, *Planctomycetes*, *Prevotella*, *Veillonella*, and many unidentified genera were enriched in patients with stage II HCC. *Actinomyces*, *Atopobium*, *Desulfococcus*, *Enterobacter*, *Haemophilus*, *Lactococcus*, *Leptotrichia*, *Neisseria*, *Oribacterium*, *Prevotella*, *Rothia*, *Selenomonas*, *Veillonella*, and many unidentified genera were multiplied in patients with stage III HCC (Figure 2B and Supplementary Figure S2). Further, *Desulfococcus*, *Enterobacter*, *Prevotella*, *Veillonella*, and many unidentified genera were increased in all stages of HCC. However, *Acidaminococcus*, *Cetobacterium*, *Coprobacillus*, *Pyramidobacter*, *Turicibacter*, and two unidentified genera were reduced in patients with stage I HCC; and *Anaerotruncus*, *Cetobacterium*, and an unidentified genus were decreased in patients with stage II HCC. Moreover, *Acidaminococcus*, *Anaerostipes*, *Anaerotruncus*, *Butyricimonas*, *Cetobacterium*, *Cloacibacillus*, *Coprobacillus*, *Holdemania*, *Methanobrevibacter*, *Odoribacter*, Pyramidobacter, Turicibacter, and four unidentified genera were reduced in patients with stage III HCC. *Cetobacterium* was reduced in all stages of primary HCC (Figure 2B and Supplementary Figure S3).

**FIGURE 3:**
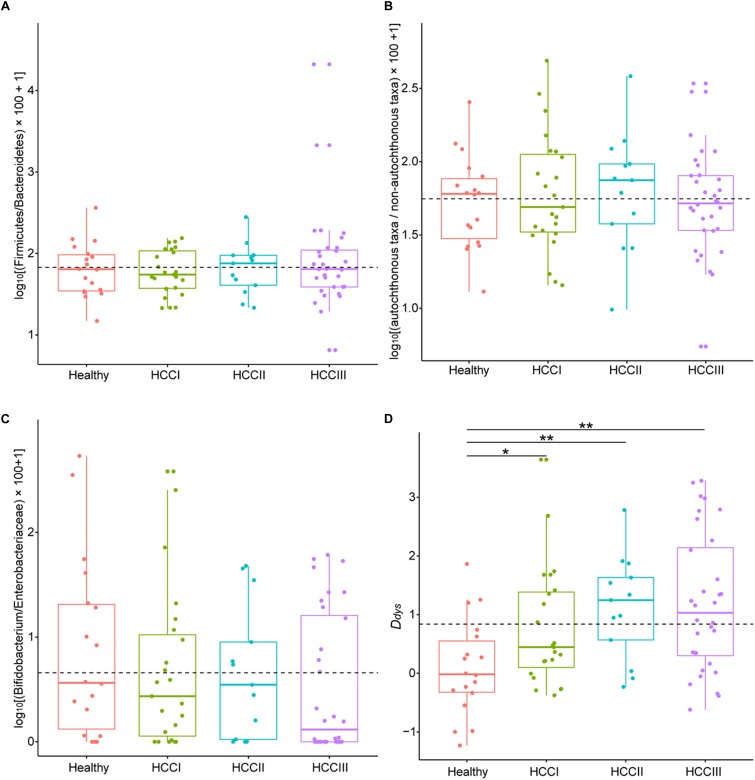
Boxplots showing changes by degree of dysbiosis. Description of indices and how they were calculated **(A)** based on the relative abundance of Firmicutes and those of Bacteroidetes, **(B)** based on the relative abundance of autochthonous taxa and those of non-autochthonous taxa, **(C)** based on the relative abundance of *Bifidobacterium* and those of Enterobacteriaceae, and **(D)** based on seven probiotic bacterial genera (*Anaerostipes*, *Bifidobacterium*, *Coprococcus*, *Faecalibacterium*, *Lactobacillus*, *Oscillibacter*, and *Phascolarctobacterium*) and 13 harmful bacterial genera (*Akkermansia*, *Bacteroides*, *Clostridium*, *Dorea*, *Escherichia*, *Fusobacterium*, *Haemophilus*, *Helicobacter*, *Klebsiella*, *Prevotella*, *Ruminococcus*, *Streptococcus*, and *Veillonella*). Primary HCC samples were staged as previously described (Zhou et al., 2018). ^∗∗^*p* < 0.01; ^∗^*p* < 0.05.

**FIGURE 4:**
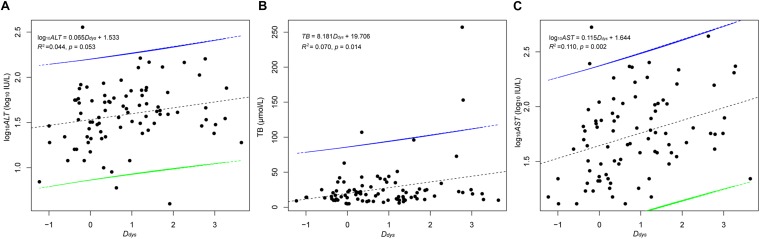
Correlation between the *D_dys_* and the ALT level **(A)**, the TB **(B)**, and the AST **(C)**. The *D_dys_* was calculated based on seven probiotic bacterial genera (*Anaerostipes*, *Bifidobacterium*, *Coprococcus*, *Faecalibacterium*, *Lactobacillus*, *Oscillibacter*, and *Phascolarctobacterium*) and 13 harmful bacterial genera (*Akkermansia*, *Bacteroides*, *Clostridium*, *Dorea*, *Escherichia*, *Fusobacterium*, *Haemophilus*, *Helicobacter*, *Klebsiella*, *Prevotella*, *Ruminococcus*, *Streptococcus*, and *Veillonella*). The black dotted lines are the correlation lines. The intervals between the green and blue lines are the confidence intervals.

The ratio of abundance of Firmicutes to Bacteroidetes (Kruskal–Wallis test, χ^2^ = 0.413, *p* = 0.938; Figure 3A), autochthonous taxa to non-autochthonous taxa (Kruskal–Wallis test, χ^2^ = 0.741, *p* = 0.864; Figure 3B), and genus *Bifidobacterium* to the family Enterobacteriaceae (Kruskal–Wallis test, χ^2^ = 2.942, *p* = 0.401; Figure 3C) revealed no significant difference between primary HCCs at different stages and the healthy controls. However, this analysis was not comprehensive so this study created a more integrated index called degree of dysbiosis (*D_dys_*) and analyzed the *D_dys_* of the gut microbiota at each primary HCC stage and in healthy controls. Among the 20 common gut bacterial genera that were used to calculate the *D_dys_*, one essentially probiotic bacterial genus (*Oscillibacter*) and two potentially harmful bacterial genera (*Akkermansia* and *Helicobacter*) were not detected in the present study. The *D_dys_* significantly increased in patients with primary HCC compared with that in healthy controls. The increase in *D_dys_* was continued, and a tendency of *D_dys_* to increase emerged with the progression of primary HCC, although no significant difference was detected between patients with different stages of primary HCC (Figure 3D). In addition, although there was no significant correlation between the *D_dys_* and the *ALT* level (log_10_*ALT* = 0.065*D_dys_* + 1.533, *R*^2^ = 0.044, *p* = 0.053; Figure 4A), the *D_dys_* positively correlated with the total bilirubin concentration (*TB* = 8.181*D_dys_* + 19.706, *R*^2^ = 0.070, *p* = 0.014; Figure 4B) and AST level (log_10_*AST* = 0.115*D_dys_* + 1.644, *R*^2^ = 0.110, *p* = 0.002; Figure 4C), which are commonly used to indicate the liver function.

## Discussion

Accumulating evidence has supported the notion that persistent inflammation leads to HCC (Darnaud et al., 2013). Pro-inflammatory factors, such as lipopolysaccharide (LPS) and flagellin, activate the nuclear factor-κB pathway, produce pro-inflammatory cytokines [tumor necrosis factor-α, interleukin-6 (IL-6), and IL-1], and lead to liver inflammatory and oxidative damage (Darnaud et al., 2013). Dysbiosis of the gut microbiota increases LPS-producing bacteria and changes bile acid metabolism. Moreover, while controversial (Darnaud et al., 2013), dysbiosis is considered a promoter of liver inflammation which could ultimately lead to HCC (Yu et al., 2010; Yu and Schwabe, 2017; Ma et al., 2018). Therefore, dysbiosis has been extensively studied in order to characterize the gut microbiome in patients with HCC, or screen non-invasive biomarkers for primary HCC, and prevent or adjunctively treat primary HCC through gut microbiota (Ren et al., 2018).

Many indices are utilized to measure dysbiosis in patients with CLDs. Lu et al. (2011) reported that the *Bifidobacterium*/Enterobacteriaceae ratio may act as an indicator of the level of dysbiosis over the course of liver disease progression. Wong et al. (2013) reported that the abundance of Firmicutes reduced and those of Bacteroidetes increased in the fecal microbiota of patients with non-alcoholic steatohepatitis, which implied that the ratio of Firmicutes to Bacteroidetes could probably be used as an indicator of non-alcoholic steatohepatitis. In addition, the ratio of autochthonous to non-autochthonous taxa was calculated as the cirrhosis dysbiosis ratio (Bajaj et al., 2014b). However, these indices did not apply to degree the dysbiosis experienced by patients with primary HCC in the present study (Figure 3A–C), and thus a new index was required. Thus, in this study, we introduced the *D_dys_* index to be utilized as a tool to measure dysbiosis. Our results showed that the *D_dys_* increased significantly in patients with primary HCC compared with that of the healthy controls. Additionally, the *D_dys_* tended to increase as the HCC stage increased, suggesting that the *D_dys_* may indicate the degree of dysbiosis in patients with primary HCC. The ratio of the abundance of *Bifidobacterium* to *Enterococcus* was proposed as a measure of pre-liver transplantation in gut dysbiosis (Grąt et al., 2015). However, more than four-fifths of the samples could not detect *Enterococcus* in the present study. Thus, we did not include this index in the present study.

The expansion of taxa from Proteobacteria, especially the family Enterobacteriaceae, was reported in many cases of dysbiosis in patients with different diseases (Shin et al., 2015; Grąt et al., 2016; Hegde et al., 2018). We also found that the relative abundance of Proteobacteria was enhanced in the present study (Figure 1C). In addition, many genera of Proteobacteria, such as *Enterobacter* and *Haemophilus*, were also increased in the fecal samples of patients with primary HCC (Figure 2B).

In the present study, the proliferated bacteria included into the calculation of the *D_dys_* were commonly associated with inflammatory bowel disease (IBD) (Png et al., 2010; Shaw et al., 2016), irritable bowel syndrome (Bhattarai et al., 2017), liver tumor development and metastases (Ma et al., 2018; Ren et al., 2018), gastric cancer (Coker et al., 2018), and colorectal cancer (Aymeric et al., 2018; Oh, 2018; Shang and Liu, 2018), which are all associated with dysbiosis. Therefore, the *D_dys_* index could potentially be used to indicated the degree of dysbiosis in patients with other diseases, such as IBD and colorectal cancer. However, further studies are needed to verify the applicability of this index in these diseases.

## Conclusion

In conclusion, we introduced the *D_dys_* index to measure dysbiosis and found that the *D_dys_* was significantly increased in patients with primary HCC compared with that of the healthy controls. Additionally, the *D_dys_* tended to increase with the development of primary HCC, although no significant difference was detected between patients with different stages of primary HCC. However, since the *D_dys_* continued to increase in the bacterial genera that were closely correlated with dysbiosis in primary HCC, further studies are needed to verify the application of this index for use in other diseases.

## Data Availability

The datasets generated for this study can be found in NCBI Sequence Read Archive database, SRP151835.

## Ethics Statement

The study protocol was approved by the Medical Ethics Committee of Zhujiang Hospital, Southern Medical University (approval number: 2017-GDEK-002) and was performed in accordance with clinical ethics guidelines and the Declaration of Helsinki and Rules of Good Clinical Practice. Patients with HCC, as well as age-matched healthy controls, were recruited from five hospitals in Guangzhou, a large modern city in southern China. All patients and healthy controls provided informed consent for participation in the study. No specific medical intervention was conducted specifically for this study.

## Author Contributions

JN, RH, XX, HZ, KZ, and YG designed the research study. JN, RH, XX, YL, KZ, MG, XC, BH, MY, BP, QL, and PZ conducted the research. JN, RH, HZ, and PC collected and analyzed the data. JN, XX, and YG wrote the manuscript. All authors approved the final version of the manuscript.

## Conflict of Interest Statement

The authors declare that the research was conducted in the absence of any commercial or financial relationships that could be construed as a potential conflict of interest.

## Supplementary Material

FIGURE S1 Percentage bar diagram showing compositions of the dominant phyla (their relative abundances were more than 1% in at least one sample) in the gut microbiota of patients with different stages of primary HCC and healthy controls.

FIGURE S2 Boxplots showing significantly enhanced taxa for the gut microbiota from patients at different stages of primary HCC compared with the healthy controls. Primary HCC samples were staged as previously described (Zhou et al., 2018). ^∗∗∗^*p* < 0.001; ^∗∗^*p* < 0.01; ^∗^*p* < 0.05.

FIGURE S3 Boxplots showing a significantly reduction in the taxa of the gut microbiota from patients at different stages of primary HCC compared with healthy controls. Primary HCC samples were staged as previously described (Zhou et al., 2018). ^∗∗∗^*p* < 0.001; ^∗∗^*p* < 0.01; ^∗^*p* < 0.05.
