# Supplementary figures and images for: Analysis of the Relationship Between the Degree of Dysbiosis in Gut Microbiota and Prognosis at Different Stages of Primary Hepatocellular Carcinoma
Source: Front Microbiol. 2019 Jun 25;10:1458. doi: 10.3389/fmicb.2019.01458 (PMC6603198; doi:10.3389/fmicb.2019.01458)

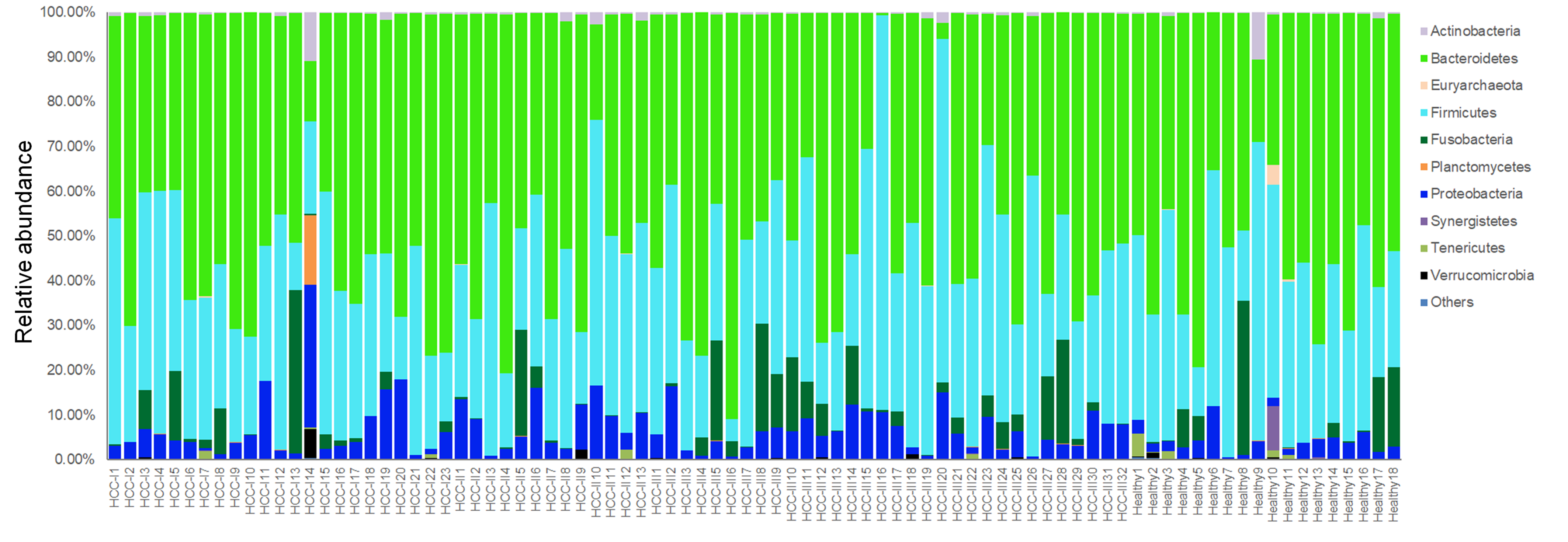

Supplement: FIGURE S1 — Percentage bar diagram showing compositions of the dominant phyla (their relative abundances were more than 1% in at least one sample) in the gut microbiota of patients with different stages of primary HCC and healthy controls. [file Image_1.TIF]

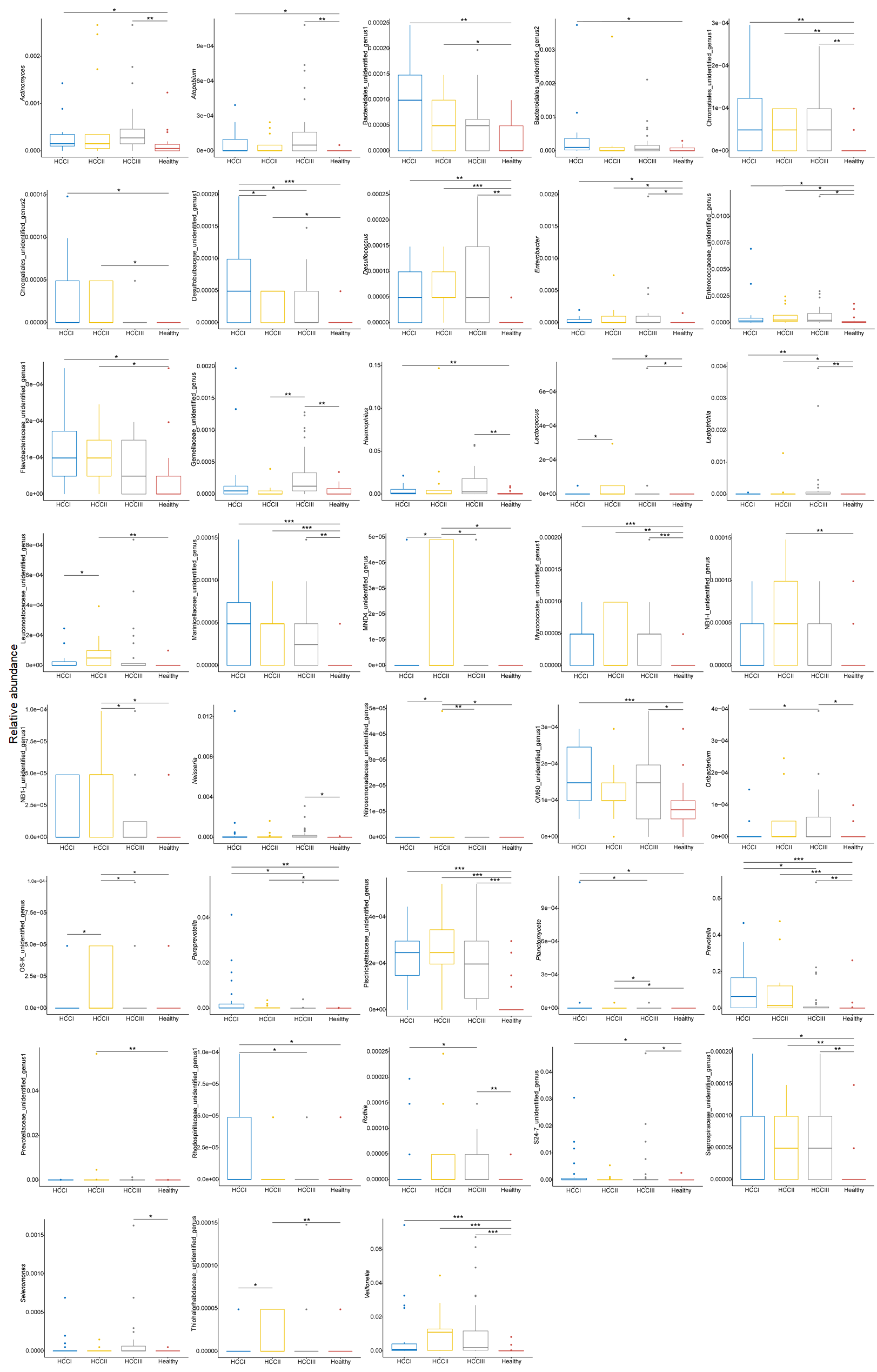

Supplement: FIGURE S2 — Boxplots showing significantly enhanced taxa for the gut microbiota from patients at different stages of primary HCC compared with the healthy controls. Primary HCC samples were staged as previously described (Zhou et al., 2018). ∗∗∗p < 0.001; ∗∗p < 0.01; ∗p < 0.05. [file Image_2.TIF]

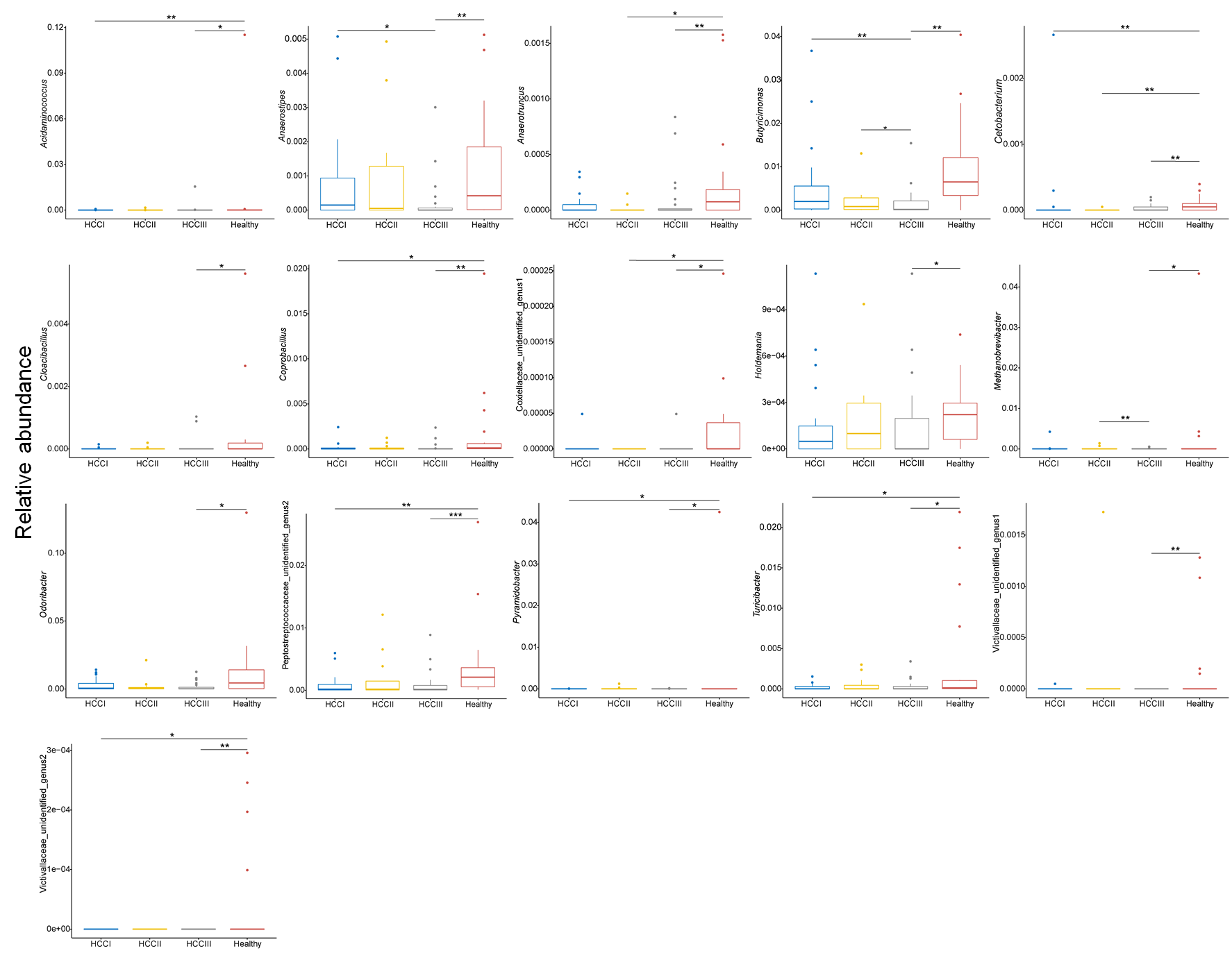

Supplement: FIGURE S3 — Boxplots showing a significantly reduction in the taxa of the gut microbiota from patients at different stages of primary HCC compared with healthy controls. Primary HCC samples were staged as previously described (Zhou et al., 2018). ∗∗∗p < 0.001; ∗∗p < 0.01; ∗p < 0.05. [file Image_3.TIF]
